# Supplementary material for: Gallium Nitrate Enhances Antimicrobial Activity of Colistin against Klebsiella pneumoniae by Inducing Reactive Oxygen Species Accumulation
Source: Microbiol Spectr. 2023 Jun 5;11(4):e00334-23. doi: 10.1128/spectrum.00334-23 (PMC10434156; doi:10.1128/spectrum.00334-23)
Supplement: Supplemental file 1 — Tables S1 and S2 and Fig. S1 to S11. Download spectrum.00334-23-s0001.pdf, PDF file, 1.1 MB [file spectrum.00334-23-s0001.pdf]

Table S1. Antimicrobial Susceptibility for the selected clinical Strains

bacterial	Lipopeptides	Carbapenems			Penicillins		Cephalosporins alkene			Monocyclic lactam	Aminoglycosides	
isolate	COL	ETP	IMP	MEM	AMP	PIP	FEP	CAZ	CFP	ATM	GEN	AMK
GN200043	0.5	>16	>32	>32	>256	>1024	64	64	>512	>128	128	512
GN200059	32	0.0625	2	0.125	>256	128	64	16	256	64	128	8
GN200774	0.5	>16	>32	>32	>256	1024	128	>128	>512	128	>128	>512
GN200999	1	>16	>32	>32	>256	1024	128	64	>512	>128	>128	256
GN201181	16	2	4	0.25	>256	1024	16	128	512	128	>128	>512
GN201286	0.5	>16	>32	>32	>256	1024	64	32	>512	>128	64	128
GN202933	16	1	2	0.0625	>256	>1024	128	64	>512	>128	>128	>512
GN190808	0.5	>16	>32	>32	>256	>1024	>128	>128	>512	>128	128	128
GN191034	>32	1	2	≤0.0625	>256	1024	32	64	512	64	128	>512
GN191097	8	0.25	0.5	≤0.0625	>256	1024	16	32	512	64	128	2
GN191324	0.5	>16	>32	>32	>256	>1024	>128	>128	>512	>128	>128	>512
GN192079	32	0.5	1	0.125	>256	>1024	64	32	>512	64	>128	>512

GN192105	1	>16	>32	16	>256	>1024	32	64	512	>128	>128	>512
GN181719	0.5	>16	>32	>32	>256	>1024	>128	128	>512	>128	>128	>512
GN182201	16	>16	>32	>32	>256	>1024	>128	>128	>512	>128	>128	>512
GN170354	16	>16	8	4	>256	>1024	>128	>128	>512	>128	>128	>512
GN171044	>32	4	2	0.5	>256	>1024	32	16	>512	128	32	4
GN172646	1	>16	>32	>32	>256	>1024	>128	64	>512	>128	>128	>512
GN172769	1	>16	>32	>32	>256	>1024	>128	>128	>512	>128	>128	>512
GN172859	16	>16	>32	>32	>256	>1024	>128	>128	>512	>128	64	128
ATCC 43816	0.5	≤0.0313	0.5	≤0.0625	64	8	≤0.125	≤0.25	≤1	≤0.25	0.5	≤1

bacterial isolate	Tetracyclines		Quinolones		Sulfonamides		Chloram phenicols	Fosfomycin class	Nitrofurans	β -lactam compound		
	DOX	MNO	CIP	LVX	SXT	TMP	CHL	FOS	NIT	AMC	SAM	TZP
GN200043	16	8	>8	16	>32	>128	>256	2048	128	>256	>256	>1024
GN200059	16	8	8	16	>32	>128	256	64	128	256	128	8
GN200774	128	128	>8	>16	>32	>128	256	512	>1024	>256	128	1024
GN200999	32	16	>8	>16	>32	>128	32	128	128	>256	>256	1024
GN201181	64	64	>8	>16	>32	>128	256	32	128	64	128	256

GN201286	32	16	>8	>16	>32	128	128	128	256	>256	>256	>1024
GN202933	128	32	>8	>16	>32	>128	>256	128	256	>256	128	512
GN190808	16	8	>8	16	>32	>128	64	128	256	>256	>256	>1024
GN191034	64	32	>8	>16	>32	>128	>256	64	256	64	128	16
GN191097	32	16	>8	>16	>32	>128	>256	64	256	16	32	4
GN191324	64	8	>8	>16	2	32	>256	1024	512	>256	>256	>1024
GN192079	>128	>128	>8	>16	>32	>128	>256	64	512	16	128	128
GN192105	32	64	>8	>16	>32	>128	64	32	128	256	>256	1024
GN181719	32	16	>8	>16	8	8	32	>2048	512	>256	>256	>1024
GN182201	128	128	>8	>16	>32	>128	16	512	256	>256	>256	>1024
GN170354	8	8	>8	>16	2	64	128	>2048	1024	256	>256	>1024
GN171044	32	8	8	16	4	128	256	512	1024	32	128	256
GN172646	8	8	>8	>16	8	>128	32	>2048	512	>256	>256	>1024
GN172769	32	8	>8	>16	>32	>128	>256	>2048	512	>256	>256	>1024
GN172859	32	16	>8	>16	>32	>128	>256	2048	1024	>256	>256	>1024
ATCC 43816	2	2	0.0625	0.125	0.25	2	8	128	64	16	16	4

MIC, minimum inhibitory concentration. COL, colistin (the range of colistin concentrations used in MIC test was 0.125 - 16 μg/mL); ETP, Ertapenem (0.0313 - 16 μg/mL); IMP,imipenem (0.0625 - 32 μg/mL); MEM, meropenem (0.0625 - 32μg/mL); AMP, ampicillin (0.5 - 256 μg/mL); PIP, piperacillin (1 - 1024 μg/mL); FEP, cefepime (0.125 - 128 μg/mL); CAZ, ceftazidime (1 - 512 μg/mL); CFP, cefoperazone (1 - 512 μg/mL); ATM, aztreonam (0.25 - 128 μg/mL); GEN, gentamicin (0.25 - 128 μg/mL); AMK, amikacin (0.125 - 16 μg/mL); DOX, doxycycline (0.25 - 128 μg/mL); MNO, minocycline (0.25 - 128 μg/mL); CIP, ciprofloxacin (0.016 - 8 μg/mL); LVX, levofloxacin (0.0313 - 16 μg/mL); SXT, trimethoprim-sulfamethoxazole (0.125 - 32 μg/mL); TMP, trimethoprim (0.5 - 128 μg/mL); CHL, chloramphenicol (0.5 - 256 μg/mL); FOS, fosfomycin (4 - 2048 μg/mL); NIT, nitrofurantoin (2 - 1024 μg/mL); AMC, amoxicillin-clavulanic acid (0.5 - 256 μg/mL); SAM, Cefoperazone-Sulbactam (0.5 - 256 μg/mL); TZP, piperacillin-tazobactam (1 - 1024 μg/mL).

Table S2. Primers used in this study

Gene	Primer	Sequence 5` - 3`
sodA	*sodA*/F	CCGCTGAAGAGCTGATTACC
	sodA/R	TTGAAGTTCTCCACGGAACC
sodB	*sodB*/F	GACCTGGCTGGTCAAAAATG
	sodB/R	GTTAGCCGCAACAAACTTCC
sodC	*sodC*/F	ATTACGATCCGCAGCATACC
	sodC/R	GACTGTCGGCCATGTTATCC
katE	*katE/F*	CGCATTGCTGACGATCAGAA
	katE/R	ATCGGCTTTAGTCAACGCAG
katG	*katG/F*	CTTCATCGTCCATCGCCATA
	katG/R	CGATGAGAAAGAGTGGCTGG
rrsE6	*rrsE6/F*	TTGACGTTACCCGCAGAAGAA
	rrsE6/R	GCTTGCACCCTCCGTATTACC

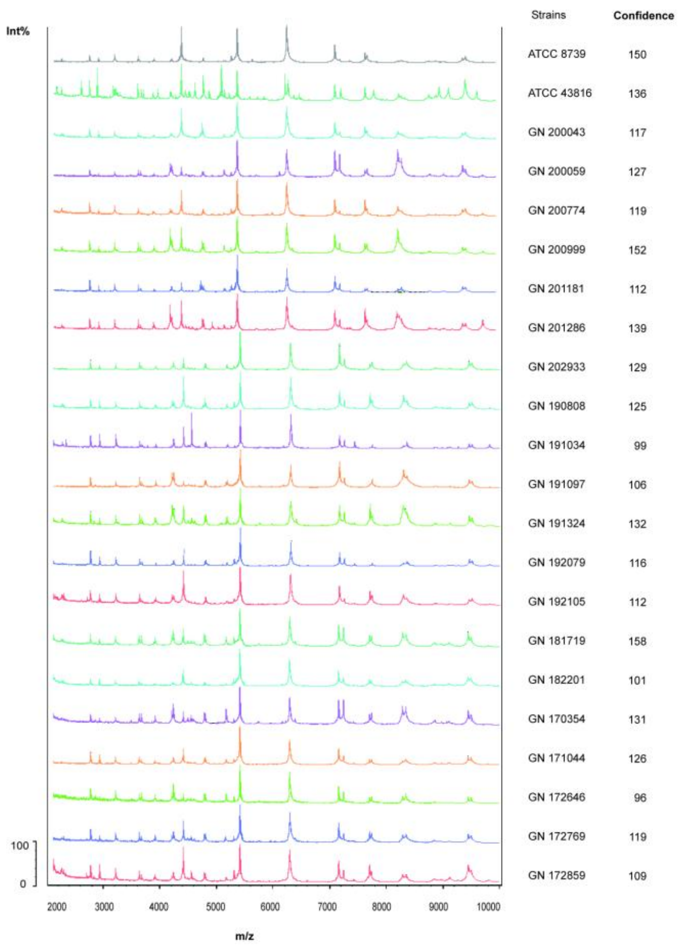

Figure S1. MALDI-TOF Mass spectrometric profiles of 20 bacterial isolate.

The measured range of 2,000 to10,000 Da is displayed. The characteristic mass peaks are predominantly ribosomal proteins. The peak of mass spectrometry of *K. pneumoniae* was 4365、5381 and 7158. Computer display of identification results after automatic comparison of the generated spectrum with the MALDI-TOF database. The degree of similarity to the reference spectrum is represented by a score value. Identification results with score values above 25 are considered to be correct for determination of the respective species.

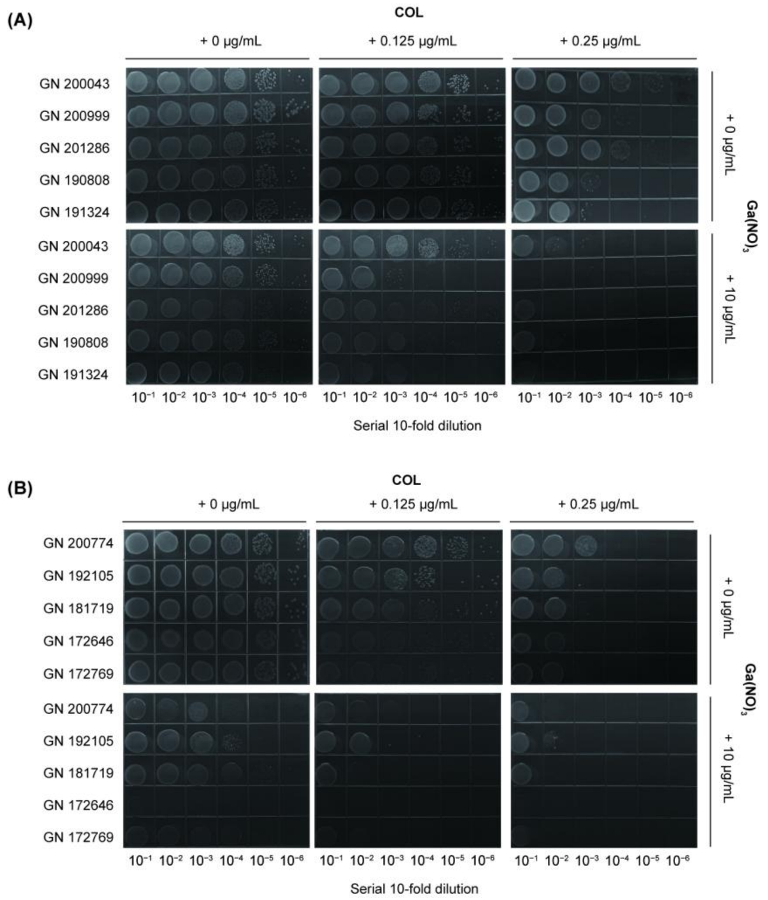

Figure S2. ***Klebsiella pneumoniae*** **sensitivity to colistin in the presence of GaNt.** **(A, B)** Growth of serial 10-fold dilutions of 10 colistin (COL)-sensitive MDR *K. pneumoniae* clinical strains on solid medium containing 0-0.25 µg/mL colistin and 0-10 µg/mL GaNt. The plates were incubated at 37 °C for 20 h and photographed. GN 200043, GN 200999, GN 201286, GN 190808, and GN 191324 were used in A, and GN 200774, GN 192105, GN 181719, GN 172646, and GN 172769 were used in B. The results are representative of three independent experiments.

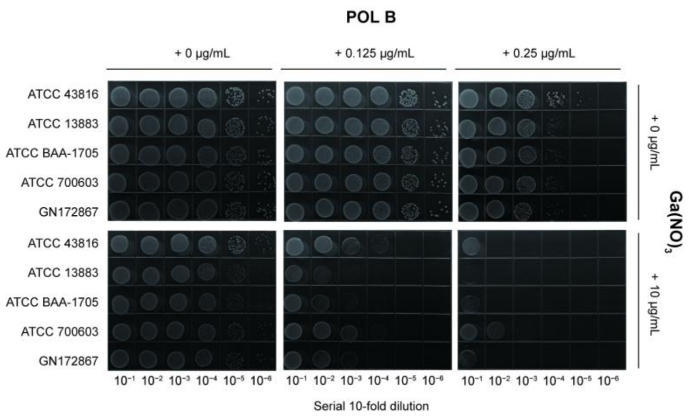

Figure S3. ***Klebsiella pneumoniae*** **sensitivity to Polymyxin B in the presence of GaNt.** Serial 10-fold dilutions of cells of 4 wild-type standard *K.pneumoniae* were spotted onto M9CA plates containing added 0 to 0.25 μg/mL Polymyxin B and 0 to 10 μg/mL GaNt. The plates were incubated at 37 °C for 20 h and photographed. The images are representative of three independent replicate experiments.

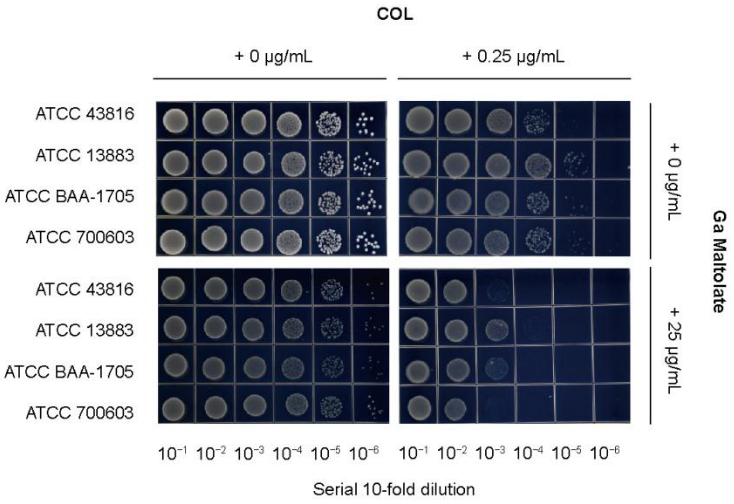

Figure S4. *Klebsiella pneumoniae* sensitivity to sensitivity to colistin in the presence of GalliuM Maltolate (Ga Maltolate). Serial 10-fold dilutions of cells of 4 wild-type standard *K.pneumoniae* were spotted onto M9CA plates containing added 0 to 0.25 μg/mL colistin(COL) and 0 to 25 μg/mL Ga Maltolate. The plates were incubated at 37 °C for 20 h and photographed. The images are representative of three independent replicate experiments.

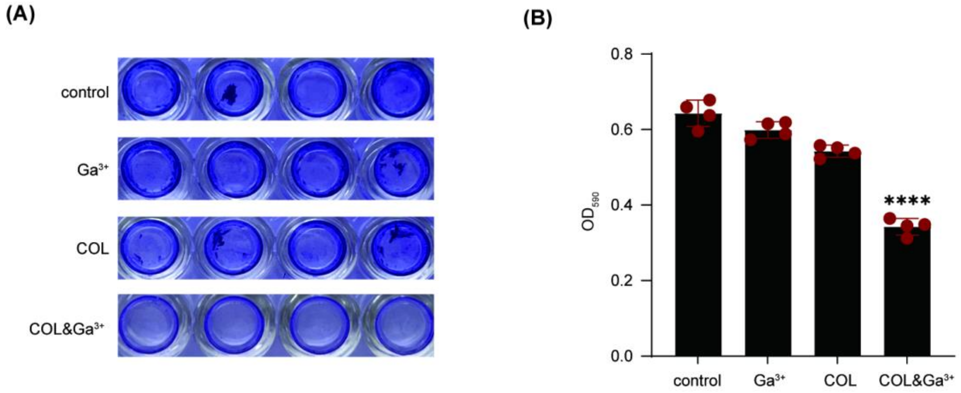

Figure S5. Effects of colistin and GaNt on *Klebsiella pneumoniae* mature biofilms. **(A)** Crystal violet staining of *K. pneumoniae* biofilms upon 2 μg/mL colistin and 20 μg/mL GaNt treatments. **(B)** Biomass quantification of biofilms in by measurement of absorbance at 590 nm. Error bars represent standard errors of the means. ****P < 0.0001 versus untreated control.

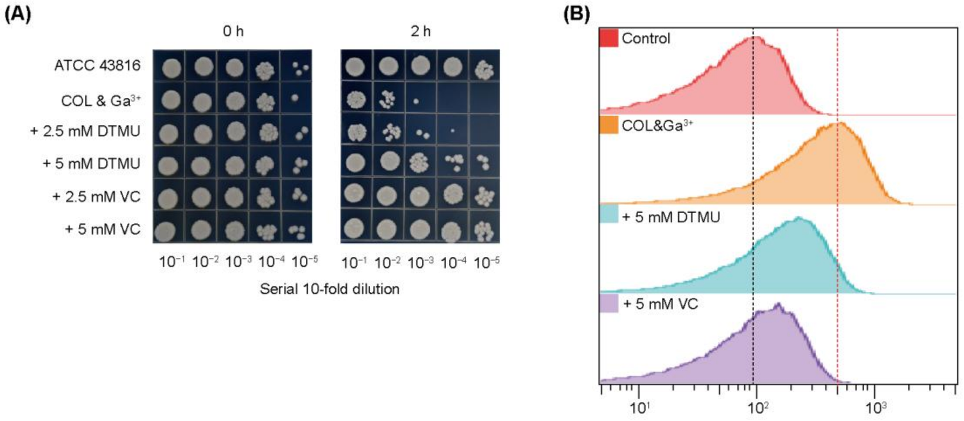

Figure S6. The effect of ROS scavenging compounds (dimethyl thiourea and ascorbic acid) on the increasement of ROS induced by GaNt-treatment. Samples were containing 2 μg/mL colistin and 20 μg/mL GaNt supplemented without or with the indicated concentrations indicated dimethyl thiourea (DMTU) or ascorbic acid (VC) for 2 h before incubation. **(A)** Serial 10-fold dilutions of samples were spotted onto M9CA plates. The plates were incubated at 37 °C for 20 h and photographed. The images are representative of three independent replicate experiments. **(B)** Bacterial ROS level were determined using flow cytometry.

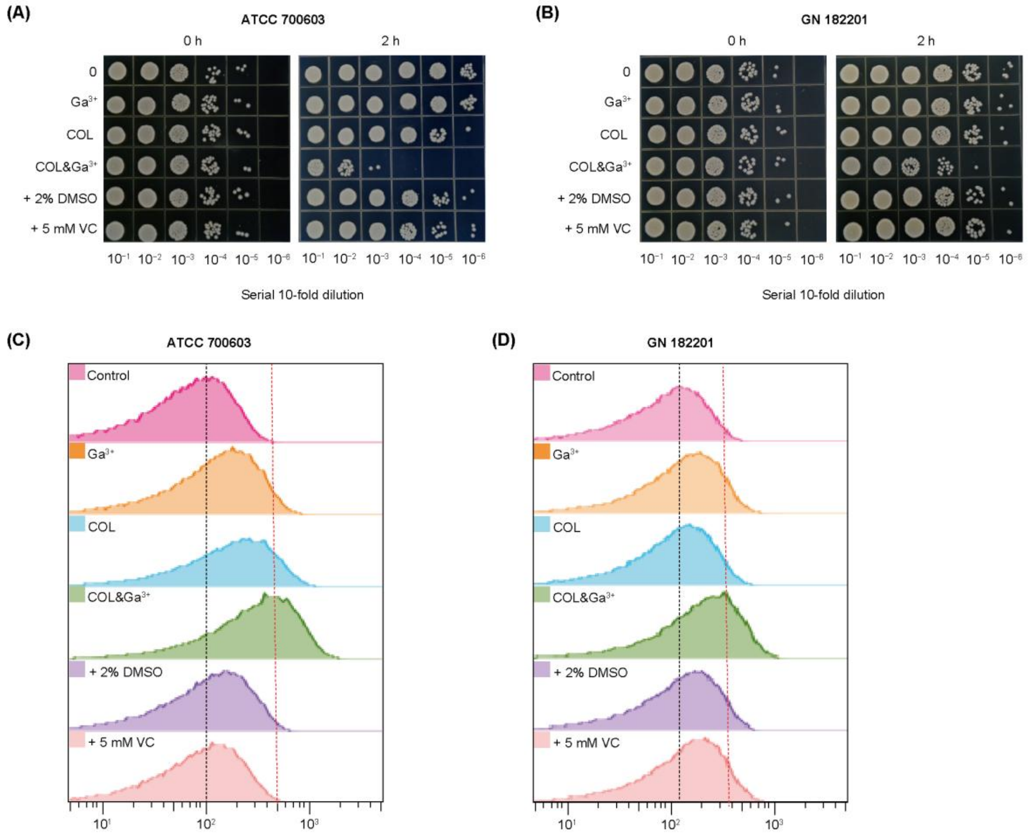

Figure S7. Rapid killing and ROS assay using *Klebsiella pneumoniae* strains ATCC 700603 and GN 182201. (A, C) *K. pneumoniae* ATCC 700603 were untreated (Control) or treated with 1 μg/mL colistin (COL), 20 μg/mL $Ga(NO_3)_3$ (Ga^{3+}), 1 μg/mL colistin and 20 μg/mL $Ga(NO_3)_3$ (COL&Ga^{3+}). Serial 10-fold dilutions of samples were spotted onto M9CA plates (A) and ROS level were determined using flow cytometry (C). (**B**, **D**) Clinical *K. pneumoniae* GN 182201 was untreated (Control) or treated with 64 μg/mL colistin (COL), 20 μg/mL $Ga(NO_3)_3$ (Ga^{3+}), 64 μg/mL colistin and 20 μg/mL $Ga(NO_3)_3$ (COL&Ga^{3+}).Serial 10-fold dilutions of samples were spotted onto M9CA plates (B) and its ROS level were determined using flow cytometry (D). All plates were incubated at 37 °C for 20 h and photographed. The images are representative of three independent replicate experiments.

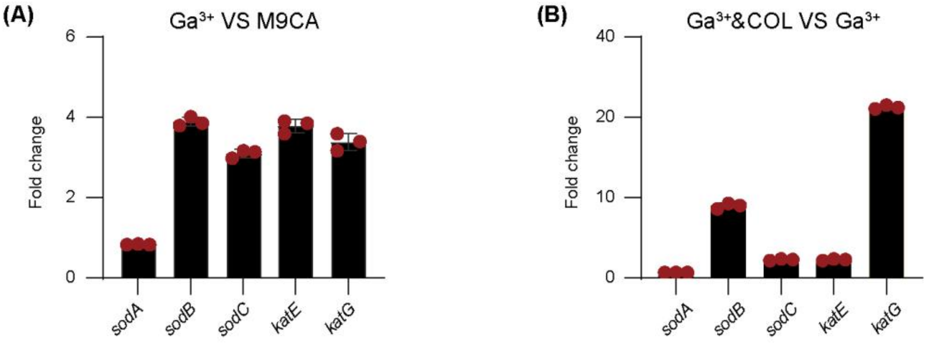

Figure S8. qRT-PCR analyses of the expression of five selected genes. ATCC 43816 were cultured for 14h and diluted 50-fold in M9CA medium without or whith GaNt **(A)** or with colistin or both **(B)**. After two-hour culture, RNA was then extracted. All assays were run in triplicate. Error bars represent standard errors of the means.

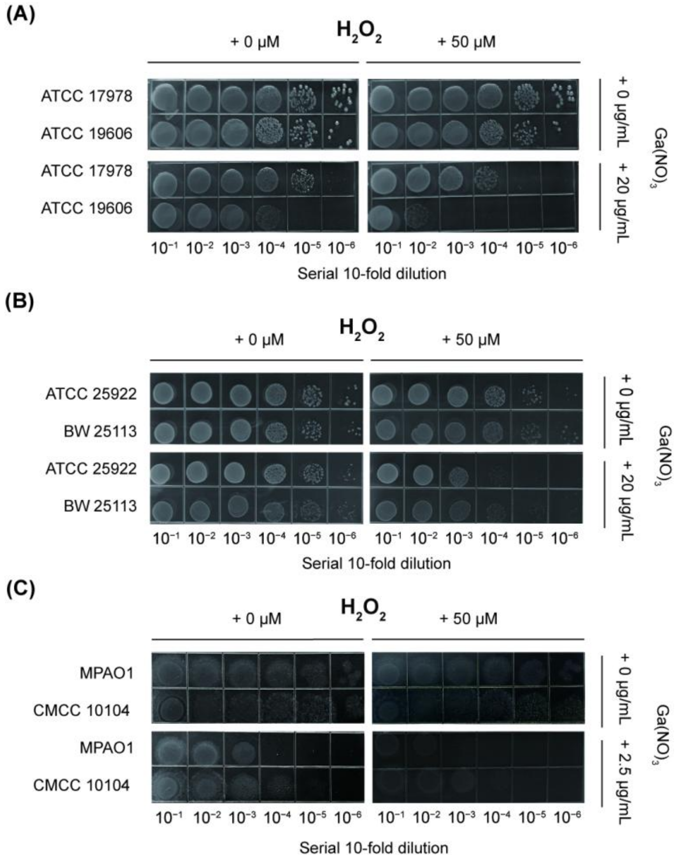

Figure S9. GaNt increased the susceptibility of *Acinetobacter baumannii*, *Escherichia coli* and *Pseudomonas aeruginosa* to H_2O_2. Serial 10-fold dilutions of cells of *A. baumannii* **(A)**, *E. coli* **(B)**, *P. aeruginosa* **(C)** were spotted onto M9CA plates containing GaNt (20 µg/ml used in *A. baumannii*, 20 µg/ml used in *E. coli*, and 2.5 µg/ml used in *P. aeruginosa*) and 0 to 50 µM H_2O_2. The plates were incubated at 37 °C for 20 h and photographed. The images are representative of three independent replicate experiments.

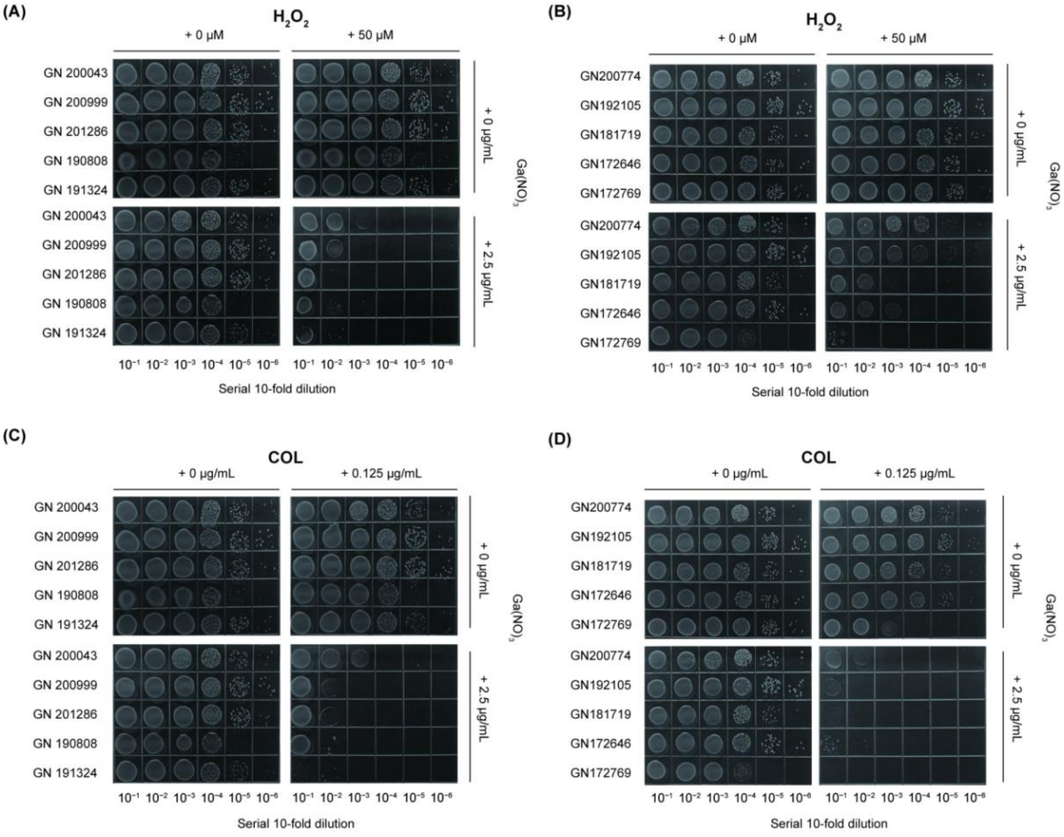

Figure S10. At the Iron-limiting conditions, GaNt increases the sensitivity of COL-sensitive *K. pneumoniae* to colistin and H_2O_2. (A, B) Growth of serial 10-fold dilutions of 10 COL-sensitive MDR clinical *K.pneumoniae* strains on M9-DIP solid medium containing 0 to 50 μM H_2O_2 and 0 to 2.5 μg/mL GaNt. **(C, D)** Samples were spotted onto M9-DIP plates containing 0 to 0.125 μg/mL colistin and 0 to 2.5 μg/mL GaNt. The plates were incubated at 37 °C for 20 h and photographed. GN 200043, GN 200999, GN 201286, GN 190808, and GN 191324 were used in (A, C), and GN 200774, GN 192105, GN 181719, GN 172646, and GN 172769 were used in (B, D). These experiments were performed on four independent occasions, and representative results are shown.

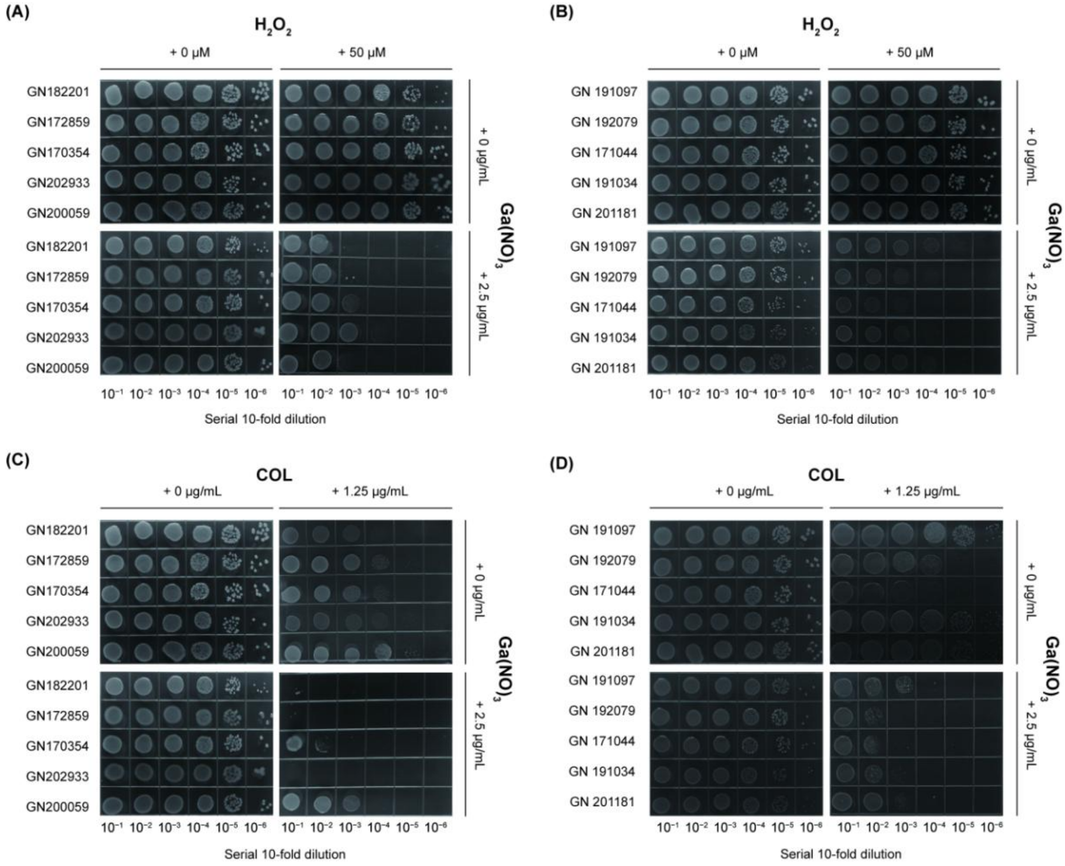

Figure S11. At the Iron-limiting conditions, GaNt increases the sensitivity of COL-resistant *K. pneumoniae* to colistin and H_2O_2. (A, B) Growth of serial 10-fold dilutions of 10 COL-resistant MDR clinical *K.pneumoniae* strains on M9-DIP solid medium containing 0 to 50 μM H_2O_2 and 0 to 2.5 μg/mL GaNt. **(C, D)** Samples were spotted onto M9-DIP plates containing 0 to 0.125 μg/mL colistin and 0 to 2.5 μg/mL GaNt. The plates were incubated at 37 °C for 20 h and photographed. GN 182201, GN 172859, GN 171044, GN 202933, and GN 200059 were used in (A, C), and GN 191097, GN 192079, GN 171044, GN 191034, and GN 201181 were used in (B, D).These experiments were performed on four independent occasions, and representative results are shown.
